# Lung Masses as a Presenting Sign of Disseminated Cryptococcus

**DOI:** 10.7759/cureus.15185

**Published:** 2021-05-22

**Authors:** Savannah Tan, George Nasr, Cameron Harding

**Affiliations:** 1 Internal Medicine, University of California Irvine, Orange, USA

**Keywords:** cryptococcosis, cryptococcus gattii, cryptococcal meningitis, disseminated cryptococcus, immunocompromised patient, pulmonary mass, neurologic complications

## Abstract

Cryptococcosis is an invasive fungal disease that most commonly affects immunocompromised individuals, typically causing pulmonary and central nervous system (CNS) symptoms. The fungus that causes cryptococcosis, *Cryptococcus, *is globally disseminated and often transmitted through bird droppings. The two most frequent and pertinent species responsible for clinical infections in humans include *Cryptococcus neoformans*, which has been known to cause the majority of cryptococcosis globally until recently, during which *Cryptococcus gattii* has been identified and reported more frequently.

A 54-year-old male with a history of renal transplant on chronic immunosuppressants and type 2 diabetes mellitus was found to have multiple lung masses within the right upper and right lower lobes. He had also been experiencing syncope, multiple falls, worsening headaches, tinnitus, diplopia, and ongoing weight loss. The patient underwent a percutaneous biopsy of one of the lung masses in addition to a lumbar puncture (LP), both of which revealed positive cryptococcus antigen confirmed to be *C. gattii*. The patient was started on amphotericin B and flucytosine to treat cryptococcal meningitis. Despite treatment, his condition continued to worsen, necessitating daily therapeutic LP and temporary placement of a lumbar drain. Once his symptoms were better managed, he was discharged from the hospital but has continued to have serial LPs outpatient while concurrently taking fluconazole to prevent reaccumulation of cerebrospinal fluid (CSF) and recurrence of symptoms.

This report describes a unique presentation of disseminated *C. gattii* infection presenting as multiple lung masses and the subsequent management of CNS cryptococcosis.

## Introduction

*Cryptococcus* is a type of invasive fungus found in the soil worldwide and is often transmitted through bird droppings. While *Cryptococcus* infection is rare in healthy individuals, those who are immunocompromised are highly susceptible to cryptococcosis, which manifests as pulmonary and neurologic symptoms. Approximately one million cases of cryptococcosis occur throughout the world annually, with an estimated 650,000 associated deaths [1]. Specifically, *Cryptococcus neoformans* and *Cryptococcus gattii* are two of the most common species of the genus that cause disease in immunosuppressed individuals. However, there has been evidence to suggest that while *C. neoformans* is known to cause death by dissemination through the central nervous system (CNS),* C. gattii *primarily causes severe pulmonary symptoms and death without dissemination [2]. Unlike the worldwide distribution of *C. neoformans*, the distribution of *C. gattii *was found to be restricted to tropical and subtropical regions, where the lowest temperatures reached in the winter still remained above freezing, with an unusually high prevalence in Southern California [3]. Here, we describe a unique case of a 54-year-old male with disseminated *C. gattii *infection that presented as multiple lung masses.

## Case presentation

**Figure 1:**
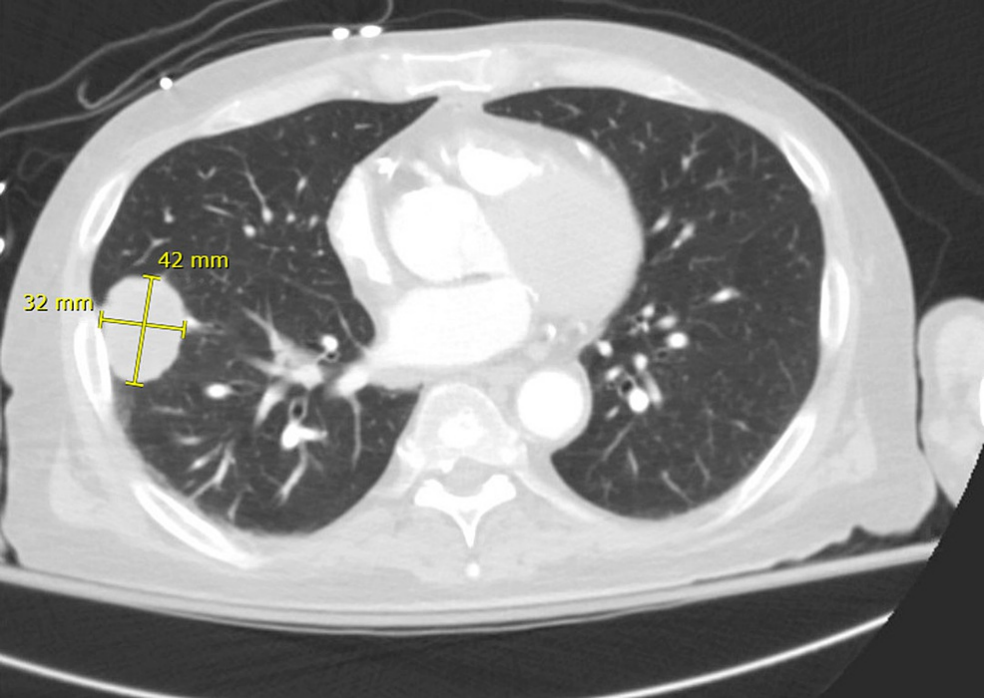
Axial view of CT chest demonstrating a right lower lobe mass in the basal lateral segment measuring 3.2 x 4.2 cm.

**Figure 2:**
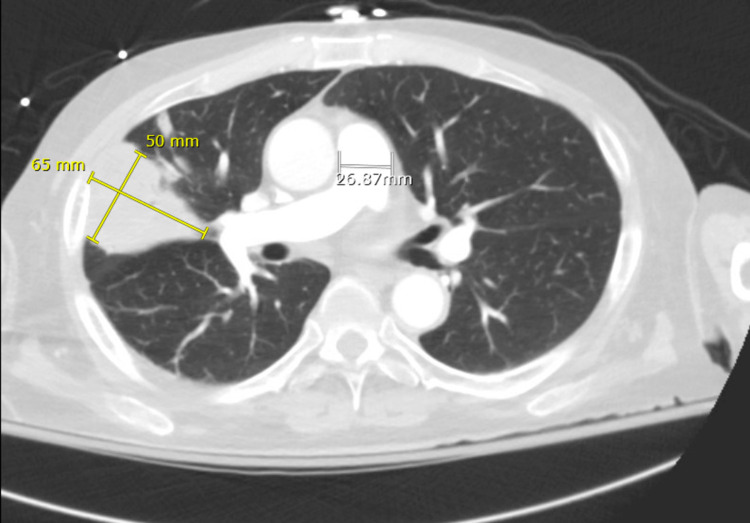
Axial view of CT chest also demonstrating a dominant mass in the right upper lobe, measuring up to 6.5 x 5.0 cm.

A 54-year-old male with a history of renal transplant one year prior to presentation due to end-stage renal disease secondary to chronic hypertension and recurrent pyelonephritis, on chronic triple agent immunosuppression including mycophenolate, prednisone, and tacrolimus, and type 2 diabetes mellitus was admitted for multiple lung masses found on chest CT (Figures 1, 2). 

He initially presented to the emergency department as trauma after a syncopal episode with brief loss of consciousness and was evaluated with a full workup that included a chest x-ray, which incidentally demonstrated round opacities in the right lower lung. CT of the chest was ordered to investigate further, and the patient was found to have pulmonary masses within the right upper and right lower lobes, the largest being 6.5 cm in the right upper lobe, concerning post-transplant malignancy such as primary bronchogenic carcinoma (Figure 2). At the time, he had been experiencing multiple syncopal episodes and falls with worsening headache, tinnitus, diplopia, and ongoing weight loss. A neurology workup was initiated to evaluate the patient’s syncope, which ruled out seizures but showed mild degree diffuse cerebral dysfunction concerning encephalopathy, with recommendations for outpatient MRI. Given his poor oral intake, the patient’s syncope was initially thought to be due to orthostatic hypotension. 

**Figure 3:**
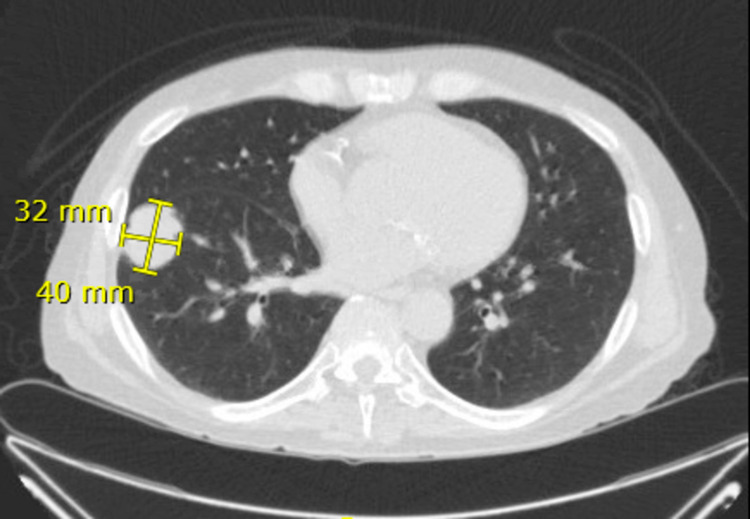
Axial view of repeat CT chest two months after initial CT, re-demonstrating right lower lobe mass in the basal lateral segment measuring 3.2 x 4.0 cm.

**Figure 4:**
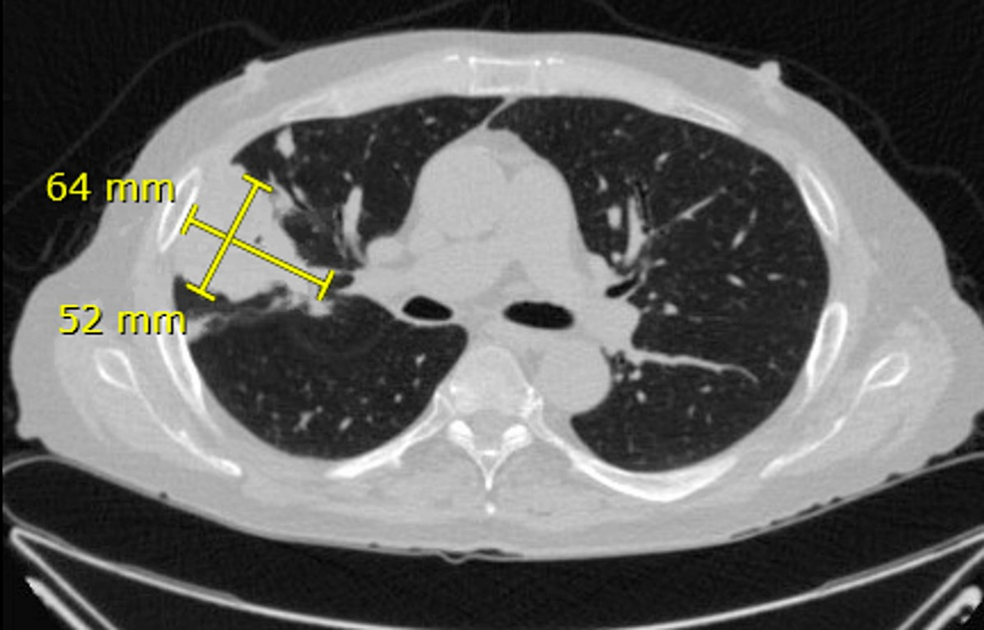
Axial view of repeat CT chest two months after initial CT, re-demonstrating dominant mass in the right upper lobe, measuring up to 6.4 x 5.2 cm.

The patient underwent a percutaneous biopsy of the lung mass to further evaluate the incidental finding. The transthoracic approach was chosen based on peripheral mass location. Preliminary results of the biopsy showed growth of yeast and likely fungal infection, so blood cultures, galactomannan, Fungitell, and serum cryptococcal, Coccidioides, and Histoplasma antigen tests were ordered. Lumbar puncture showed an opening pressure greater than 50 cm H_2_O, lymphocytic pleocytosis, and a positive cryptococcus antigen. Lung biopsy results were finalized as *C. gattii,* confirmed with positive serum antigen. The patient was started on amphotericin B and flucytosine to treat cryptococcal meningitis, and his immunosuppression regimen was adjusted to decrease over suppression by discontinuing mycophenolate sodium. Despite treatment, he continued to have worsening headaches, tinnitus, and diplopia. Daily therapeutic lumbar punctures (LPs) were performed to treat his ongoing symptoms but the opening pressure failed to decrease during subsequent LPs. A lumbar drain was subsequently placed for continuous cerebrospinal fluid (CSF) removal. He continued to receive induction therapy with amphotericin B and flucytosine for disseminated* C. gattii* infection until resolution of infection, confirmed with negative CSF cultures, after which he was transitioned to oral fluconazole. The lumbar drain was removed following lower drainage rates. His most recent chest CT, two months after initial presentation, showed continued consolidative opacities in the peripheral right lung consistent with cryptococcal infection, without significant change in size (Figures 3, 4). He is scheduled for a repeat chest CT to be performed seven months after the initial presentation. Currently, the patient’s mental and neurological status is back to baseline with plans to follow-up with neurosurgery, infectious disease, and nephrology.

## Discussion

*Cryptococcus* is dispersed throughout the environment, often associated with bird droppings or soil, enters the human body by inhalation, and in most cases is eliminated by the host’s defense mechanisms [4]. However, under certain circumstances, such as in hosts with HIV/AIDS, patients taking immunosuppressive medications, or those who have undergone solid organ transplant, the infection instead progresses to pneumonia and subsequently disseminates throughout the host body and CNS, causing meningitis or meningoencephalitis and death [5]. Numerous studies have shown that *C. neoformans* grows quickly in the brain and more commonly manifests with meningitis or meningoencephalitis while *C. gattii* infection customarily presents as a pulmonary disease because of its ability to grow faster in the lungs [6]. The presentation of this patient follows the same trend, as he presented first for and workup was initiated to evaluate multiple lung masses found on CT but was later also found to have meningitis. Initially, the patient was assumed to be experiencing headaches caused by possible lung malignancy. However, once the biopsy revealed a fungal cause, a serum cryptococcal antigen was immediately checked. This patient also possesses risk factors that made him more susceptible to infection by *Cryptococcus*, including the history of a solid organ transplant and immunosuppression. Cryptococcosis is usually seen in a transplant recipient at least six months after transplantation, with an asymptomatic pulmonary nodule containing the organism being the most common presentation of infection [7]. Infection by *C. neoformans *or *C. gattii *should be suspected in transplant patients who present more than six months after transplantation with a pulmonary nodule, unexplained headaches, decreased levels of consciousness, failure to thrive, or unexplained focal dermatologic disease. Elevated titers of cryptococcal antigen in the CSF, positive CSF culture, and high CSF pleocytosis are markers of high fungal burden and severe inflammation in cryptococcosis [5]. The etiology of elevated intracranial pressure has been theorized to be due to parenchymal inflammation, interstitial edema, and decreased outflow of CSF due to the presence of *Cryptococcus* and its capsular polysaccharide at the arachnoid granulations [8].

Culture (e.g., blood, CSF) is the gold standard for diagnosing cryptococcal infections. *C. gattii* is generally the more aggressive cryptococcal species with a mortality rate in transplant patients of up to 20-42% [9]. In regards to management, efforts should focus on therapeutic procedures to decrease intracranial hypertension in addition to antifungal therapy with amphotericin B and flucytosine. Because intracranial hypertension correlates with poor clinical outcomes, aggressive management with serial lumbar punctures is necessary until pressures are less than 25 cm of water, with progression to lumbar drains, ventriculoperitoneal (VP) shunt, or ventriculostomy if pressures remain elevated [5]. Once the induction phase of approximately six to eight weeks of treatment with amphotericin B and flucytosine has lapsed, patients should be transitioned to oral fluconazole therapy for an average of six months or more [5]. Antifungals alone are not sufficient to treat *C. gattii* infections, and source control (i.e., lobectomy for lung infections or VP shunt for CNS involvement) is often required for complete resolution. According to the Infectious Diseases Society of America (IDSA), CNS and disseminated disease with large and multiple cryptococcomas caused by *C. gattii* infection should be treated with a combination of amphotericin B and flucytosine therapy for four to six weeks, followed by fluconazole for six to 18 months, depending on whether surgery was performed [10]. In this case, surgery such as wedge resection or segmentectomy should be considered if there is compression of vital structures, failure to reduce the size of the cryptococcoma after four weeks of therapy, or failure to thrive. In immunosuppressed patients, management should be undertaken to reduce the immunosuppression regimen. Alternatively, in severe pulmonary disease due to* Cryptococcus* in the absence of extrapulmonary or disseminated disease, patients should be treated with induction therapy of amphotericin plus flucytosine for at least two weeks, followed by fluconazole for six to 12 months [10]. Prompt recognition of disease and initiation of antifungal therapy with induction and maintenance strategies is necessary to effectively treat and manage cryptococcosis caused by *C. neoformans* and *C. gattii.*

## Conclusions

Although *C. neoformans* and *C. gatti*i are distributed widely in the environment mainly in bird droppings and soil, most immunocompetent hosts will clear the infection without developing symptoms or develop only mild cough with dyspnea. However,* Cryptococcus* is an opportunistic infection and causes disseminated and more severe symptoms in immunocompromised patients, such as those who are HIV positive, those with malignancies, or those on high-dose steroid therapy. Disseminated disease can manifest as fever, pneumonia, meningitis, and as exhibited in this patient’s presentation, pulmonary masses. *Cryptococcus *should be suspected and a blood or CSF culture should be initiated to evaluate patients presenting with these symptoms and who are at risk for disseminated disease. If confirmed, therapy should be aimed at decreasing intracranial pressures to below 25 cm of water to prevent severe clinical outcomes, with concurrent and adequate antifungal therapy. Disseminated* Cryptococcus* should be suspected in patients who are immunocompromised and present with pulmonary and/or neurological manifestations.
